# Insights into the Therapeutic Potential of Active Ingredients of *Citri Reticulatae Pericarpium* in Combatting Sarcopenia: An In Silico Approach

**DOI:** 10.3390/ijms252111451

**Published:** 2024-10-25

**Authors:** Amin Ullah, Yacong Bo, Jiangtao Li, Jinjie Li, Pipasha Khatun, Quanjun Lyu, Guangning Kou

**Affiliations:** 1 Department of Nutrition and Food Hygiene, School of Public Health, Zhengzhou University, Zhengzhou 450001, China; 2 Centre for Nutritional Ecology and Centre for Sport Nutrition and Health, Zhengzhou University, Zhengzhou 450001, China

**Keywords:** *Citri Reticulatae Pericarpium*, sarcopenia, network pharmacology, molecular dynamics simulation, molecular docking

## Abstract

Sarcopenia is a systemic medical disorder characterized by a gradual decline in muscular strength, function, and skeletal muscle mass. Currently, there is no medication specifically approved for the treatment of this condition. Therefore, the identification of new pharmacological targets may offer opportunities for the development of novel therapeutic strategies. The current in silico study investigated the active ingredients and the mode of action of *Citri Reticulatae Pericarpium* (CRP) in addressing sarcopenia. The active ingredients of CRP and the potential targets of CRP and sarcopenia were determined using various databases. The STRING platform was utilized to construct a protein–protein interaction network, and the key intersecting targets were enriched through the Kyoto Encyclopedia of Genes and Genomes (KEGG) and Gene Ontology (GO) analyses. Molecular docking was used to determine the binding interactions of the active ingredients with the hub targets. The binding affinities obtained from molecular docking were subsequently validated through molecular dynamics simulation analyses. Five active ingredients and 45 key intersecting targets between CRP and sarcopenia were identified. AKT1, IL6, TP53, MMP9, ESR1, NFKB1, MTOR, IGF1R, ALB, and NFE2L2 were identified as the hub targets with the highest degree node in the protein–protein interaction network. The results indicated that the targets were mainly enriched in PIK3-AKT, HIF-1, and longevity-regulating pathways. The active ingredients showed a greater interaction affinity with the hub targets, as indicated by the results of molecular docking and molecular dynamics simulations. Our findings suggest that the active ingredients of *Citri Reticulatae Pericarpium*, particularly Sitosterol and Hesperetin, have the potential to improve sarcopenia by interacting with AKT1 and MTOR proteins through the PI3K-AKT signaling pathway.

## 1. Introduction

Sarcopenia is a progressive disorder that leads to a gradual decrease in skeletal muscle mass, function, and strength by affecting the skeletal muscles [1]. It is widely accepted that skeletal muscle mass and regenerative ability decrease as people age, and the increasing prevalence of skeletal muscle diseases in the aging population poses a significant threat to public health [2]. One of the significant health concerns of this condition among the elderly population is the occurrence of fractures, extended hospitalization, reduced functionality, and even mortality [3]. Sarcopenia is prevalent among individuals aged 60–70, with a prevalence ranging from 5% to 13%, whereas those aged 80 years or older have a prevalence of 11–50% [4]. As far as the treatment of sarcopenia is concerned, the current management strategies emphasize pharmacological interventions [5], nutrition interventions [5], and exercise interventions [6]. However, there is no authorized medication available for the treatment of sarcopenia to date [7]. A more comprehensive understanding of the molecular mechanisms, pathogenesis, and identification of sarcopenia will contribute to its prevention. Therefore, the discovery of new pharmacological targets could offer opportunities for the development of new therapeutic approaches.

Herbal medicines, an emerging field in modern pharmacological research, offer a significant resource for assessing natural compounds with potential therapeutic effects and minimal toxicity [8]. Natural compounds have received significant attention due to their defined antioxidant properties and health benefits. Particularly, oranges, limes, grapefruits, lemons, and mandarins are notable fruits categorized within the citrus genus belonging to the Rutaceae family [9]. *Citri Reticulatae Pericarpium* is one of these natural products derived from the desiccated pericarp of the mature fruit of Citrus reticulata Blanco. It is a member of the citrus genus and the Rutaceae family. The product, widely known as tangerine peel, is distributed worldwide and is frequently referred to as Chenpi in Chinese [10,11]. The main active ingredients found in *Citri Reticulatae Pericarpium*, as determined by a previous study, are the flavonoids Naringenin, Citromitin, Hesperetin, Sitosterol, and Nobiletin [12]. These active ingredients are well known for their antioxidant and anti-inflammatory activities, as well as possessing potential and diverse health benefits, including antiviral and antimicrobial activities, antiglycemic and antidiabetic properties, and anticancer and antimutagenic capabilities [13]. Previous studies have demonstrated the hypolipidemic, anti-inflammatory, anticarcinogenic, antiatherosclerosis, and antioxidant activities of *Citri Reticulatae Pericarpium* [14,15].

Sarcopenia is characterized by a gradual decline in mass, strength, and function of skeletal muscle, whereas muscle mass and functionality decrease with advancing age [16]. Hence, skeletal muscle health is of great importance in the context of sarcopenia. The decline in skeletal muscle mass is associated with aging results from a reduction in myofiber quantity and the atrophy of individual myofibers [17]. A flavonoid, Naringin, found in *Citri Reticulatae Pericarpium,* has been reported to enhance myofiber remodeling in mice [18]. One of the important indicators of aging is mitochondrial dysfunction. The flavonoid Hesperetin has been found to increase longevity and slow down aging [19]. Nobiletin enhances mitochondrial respiration in skeletal muscle, supporting healthy aging in the context of metabolic problems [20]. Whereas the bioactive compound Tangeretin increases mitochondrial biogenesis in skeletal muscle [21]. The potential contribution of bioactive compounds of *Citri Reticulatae Pericarpium* in skeletal muscle health promotion is evident in these studies. However, the existing scientific literature does not provide a definitive understanding regarding the potential involvement of *Citri Reticulatae Pericarpium* and its active compounds in mitigating sarcopenia.

**Figure 1:**
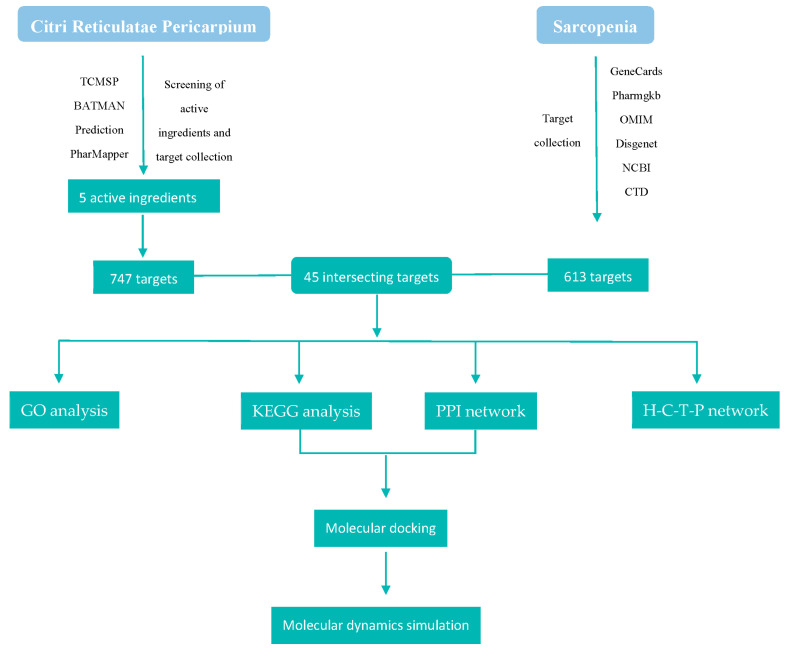
Flowchart of the study. TCMP: Traditional Chinese Medicine Systems Pharmacology; BATMAN: Bioinformatics Annotation daTabase for Molecular mechANism; OMIM: Online Mendelian Inheritance in Man; NCBI: National Center for Biotechnology Information; CTD: Comparative Toxicogenomics Database; GO: Gene Ontology; KEGG: Kyoto Encyclopedia of Genes and Genomes; PPI: protein–protein interaction; H-C-T-P: herb-compound-target-pathway.

Network pharmacology is a systematic approach designed for establishing the relationships among drugs, targets, and diseases, thereby improving the comprehension of drug pharmacology and its effects on biological networks [22]. The functional mechanisms of traditional Chinese medicine (TCM) have been comprehensively analyzed using network pharmacology. Additionally, it allows for identifying the active compounds of natural medicines that are effective in treating various diseases [23]. The objective of network pharmacology is to establish a multi-level network through various methods, including high-throughput genomics data mining and database collection. This approach aims to provide a scientific foundation for the discovery of TCM and to elucidate the principles and mechanisms of drug–body interactions at the biomolecular level [24], whereas molecular docking is a biomolecular simulation methodology that utilizes integrative bioinformatic analysis to assess the interactions between molecules. It predicts binding mechanisms and affinity at a molecular level through the application of computer programming. Considering the dynamic characteristics of proteins, it is logical to utilize powerful computing techniques, such as molecular dynamics simulations, to examine their conformation and conformational alterations in response to variations in the external environment [25]. Therefore, in the present study, we adopted a trio approach of network pharmacology, molecular docking, and molecular dynamics simulations to investigate the therapeutic effect of the active ingredients of *Citri Reticulatae Pericarpium* against sarcopenia. The schematic chart of the study is shown in Figure 1.

## 2. Results

A combination of 22 software and databases was used in the current work to investigate the active ingredients of *Citri Reticulatae Pericarpium* and their therapeutic potential in addressing sarcopenia (Table 1).

### 2.1. Active Ingredients of Citri Reticulatae Pericarpium and Associated Targets

**Figure 2:**
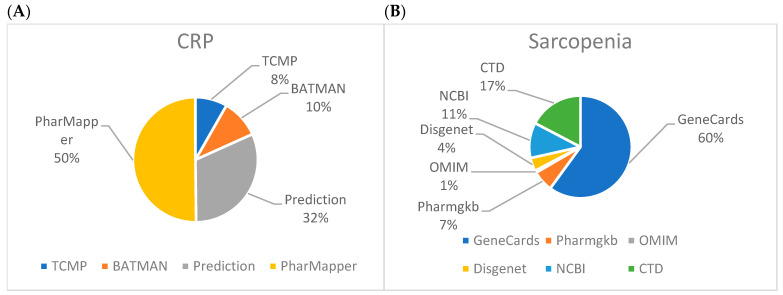
Target collection from various drug and disease databases. (**A**) Active ingredients of *Citri Reticulatae Pericarpium*-related targets; (**B**) Sarcopenia-related targets.

Five active ingredients were identified for *Citri Reticulatae Pericarpium* in the Traditional Chinese Medicine Systems Pharmacology (TCMSP) database, and 67 associated targets were retrieved. More potential targets for these ingredients were retrieved from the PharmMapper, Prediction, and Bioinformatics Annotation daTabase for Molecular mechANism of Traditional Chinese Medicine (BATMAN-TCM) databases, with 406, 255, and 81 targets, respectively (Figure 2A). The collected targets from their respective databases were combined. The elimination of 61 duplicate targets resulted in finalizing 747 potential targets. The active ingredients of *Citri Reticulatae Pericarpium* are summarized in Table 2.

### 2.2. Sarcopenia-Related Targets

A cumulative total of 430 targets were retrieved from the GeneCards database, followed by the identification of 124, 80, 47, 30, and 5 targets in the Comparative Toxicogenomics Database (CTD), National Center for Biotechnology Information (NCBI), PharmGKB, DISGENET, and Online Mendelian Inheritance in Man (OMIM), respectively. After merging the targets, 103 duplicates were identified and accordingly eliminated. A total of 613 prospective targets were confirmed for sarcopenia (Figure 2B).

### 2.3. Key Intersecting Targets of Citri Reticulatae Pericarpium and Sarcopenia

**Figure 3:**
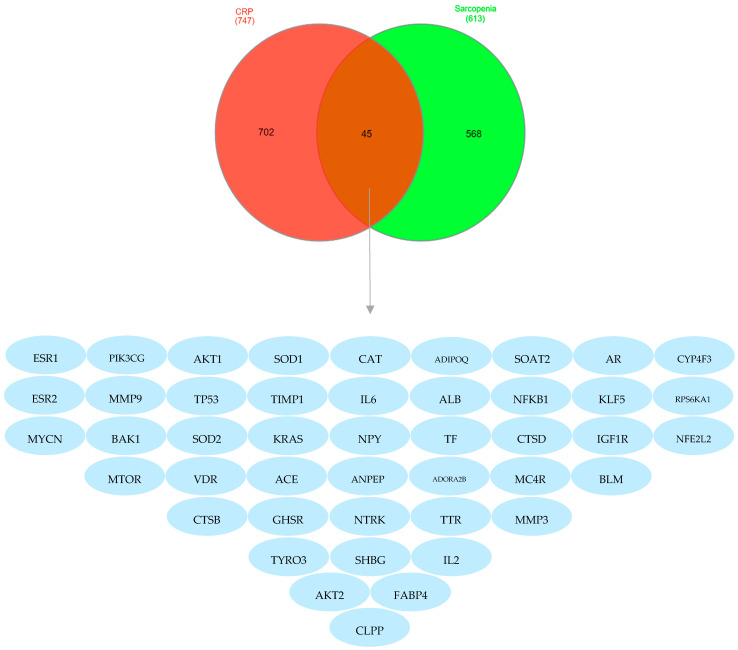
Venn diagram of *Citri Reticulatae Pericarpium* and sarcopenia: the red part represents *Citri Reticulatae Pericarpium* with 702 targets, the green part represents sarcopenia with 568 targets, while the brown intersecting part represents the core intersecting targets between *Citri Reticulatae Pericarpium* and sarcopenia.

The result of the Venn diagram revealed 45 key genes that intersected, as illustrated in Figure 3. The treatment of sarcopenia may be significantly influenced by these key intersected genes.

### 2.4. The Intersecting Key Targets and Analysis of Protein–Protein Interaction Network

**Figure 4:**
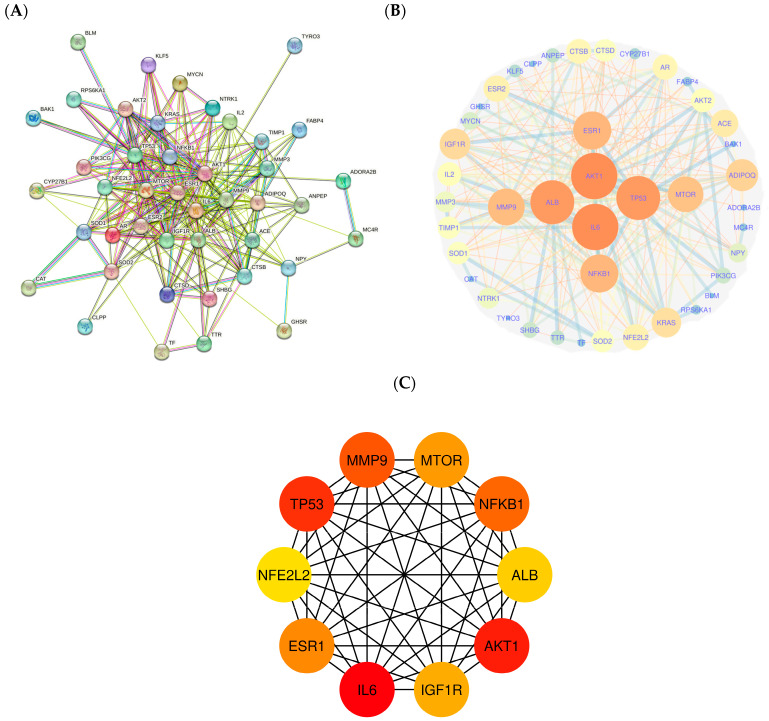
(**A**) Protein–protein interaction network of key intersecting targets of *Citri Reticulatae Pericarpium* and sarcopenia; (**B**) visualized nodes and edges of protein–protein interaction network; (**C**) top 10 hub genes in the protein–protein interaction network.

The STRING online platform was utilized to construct the protein–protein interaction network by importing the 45 key intersecting targets (Figure 4A). The protein–protein interaction network generated by the STRING platform consisted of 45 nodes, showing an average node degree of 11.9 and covering 267 edges. The degree values of a node represent its magnitude, with larger nodes indicating higher degrees. The thickness of the connecting line indicates the degree of convergence between these nodes (Figure 4B). The network was subsequently examined and visualized using Cytoscape 3.9.1 to identify the hub genes. The top 10 hub genes were analyzed using the “cytoHubba extension” within Cytoscape. The analysis identified AKT1, IL6, TP53, MMP9, ESR1, NFKB1, MTOR, IGF1R, ALB, and NFE2L2 as the top 10 hub genes (Figure 4C).

### 2.5. Analysis of Gene Ontology and Kyoto Encyclopedia of Genes and Genomes Enrichment of Key Intersecting Targets

**Figure 5:**
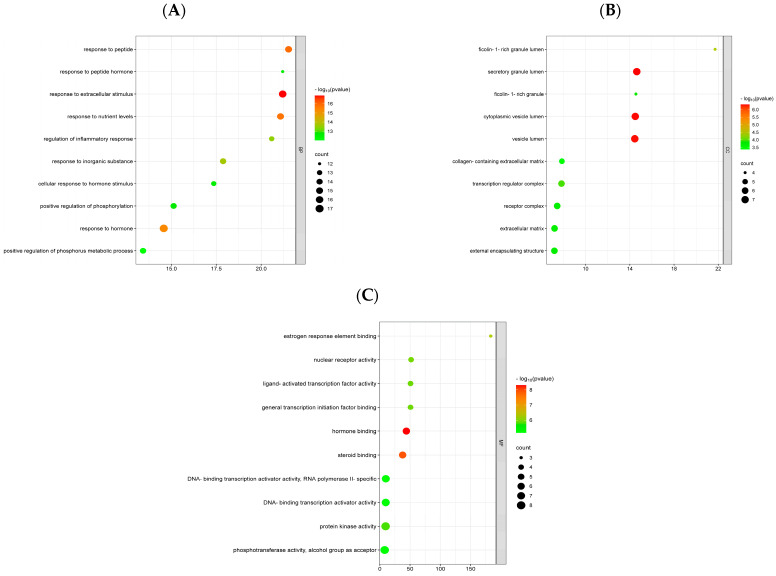

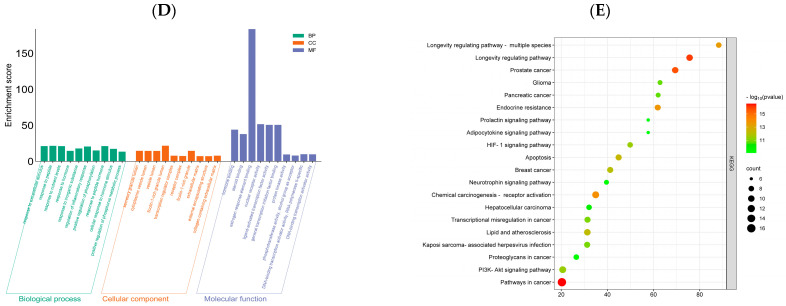
Gene Ontology (GO) and Kyoto Encyclopedia of Genes and Genomes enrichment. (**A**) Biological process (BP); (**B**) cellular component (CC); (**C**) molecular function (MF); (**D**) gene ontology bar chart; (**E**) Kyoto Encyclopedia of Genes and Genomes (KEGG) enrichment.

The Metascape platform calculated the gene ontology of 45 key intersecting targets to identify the primary activities of these targets. A total of 641 Gene Ontology terms were significantly enriched (*p* < 0.01), including 566 biological processes, 21 cellular components, and 54 molecular functions terms. Furthermore, significant enrichment was detected in the 117 Kyoto Encyclopedia of Genes and Genomes pathways. Gene ontology offers a comprehensive collection of information regarding the functions of genes and their products, including biological processes, cellular components, and molecular functions, whereas genes and genomes are categorized in the Kyoto Encyclopedia of Genes and Genomes based on their molecular and higher-order functional significance [26]. The findings of the enrichment analysis indicated that the top ten terms of biological processes were the responses to peptide, peptide hormone, extracellular stimulus, nutrient levels, inorganic substance, hormone, hormone stimulus, regulation of inflammatory response, and positive regulations of phosphorylation and metabolic process of phosphorus (Figure 5A). The enriched cellular components terms were ficolin-1-rich granule, ficolin-1-rich granule lumen, secretory granule lumen, vesicle lumen, cytoplasmic vesicle lumen, extracellular matrix, collagen-containing extracellular matrix, receptor complex, transcription regulator complex, and external encapsulating structure (Figure 5B). On the other hand, the activities of protein kinase, phosphotransferase, nuclear receptor, ligand-activated transcription factor, DNA-binding transcription activator, bindings of hormone, steroid, estrogen response element, general transcription initiation factor, alcohol group as acceptor, and RNA polymerase II-specific were the representative molecular functions terms in the enrichment analysis (Figure 5C). Figure 5D illustrates the combined gene ontology results presented in a bar chart.

**Figure 6:**
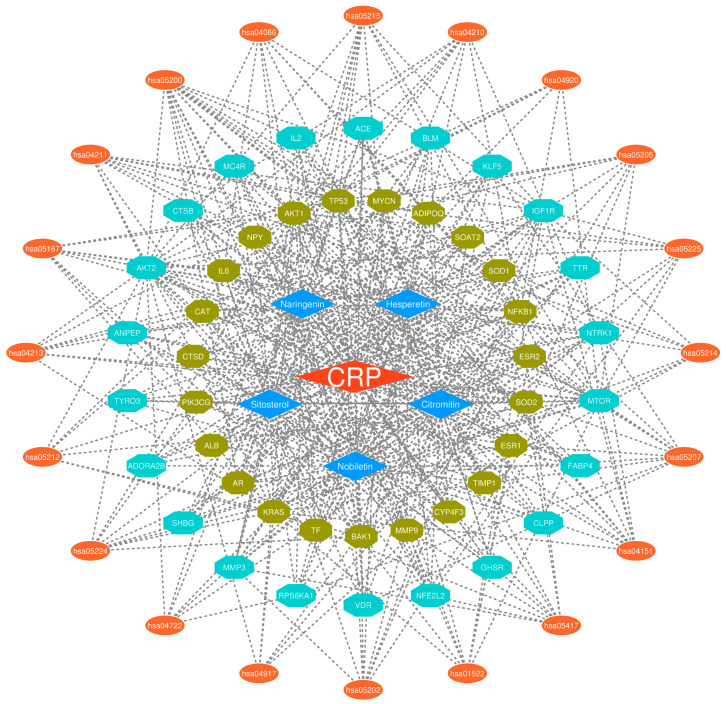
The herb-compound-target-pathway network. The orange diamond in the center represents the herb *Citri Reticulatae Pericarpium*, the five blue diamonds represent the active ingredients, the olive-green octagons and cyan octagons represent the core intersecting targets, while the outer ellipse shapes represent the pathways in the network.

In addition, the identified top 20 Kyoto Encyclopedia of Genes and Genomes terms in the network were prostate cancer, glioma, pancreatic cancer, endocrine resistance, apoptosis, breast cancer, chemical carcinogenesis-receptor activation, hepatocellular carcinoma, transcriptional misregulation in cancer, lipid, and atherosclerosis, Kaposi sarcoma-associated herpes virus infection, proteoglycans in cancer, the signaling pathways including the longevity regulating of multiple species, longevity regulating, prolactin, adipocykine, HIF-1, neurotrophin, PI3K-Akt, and pathways in cancer, respectively (Figure 5E). The findings of gene ontology and Kyoto Encyclopedia of Genes and Genomes enrichment suggest that the mitigation of sarcopenia by *Citri Reticulatae Pericarpium* may be substantially mediated by these targets and pathways. The herb-compound-target-pathway network was created using Cytoscape Software 3.9.1, as shown in Figure 6. This network demonstrates the potential of active ingredients in *Citri Reticulatae Pericarpium* to interact with several targets and pathways, suggesting that the active ingredients of *Citri Reticulatae Pericarpium* may have a significant impact on mitigating sarcopenia by targeting these specific targets and pathways.

### 2.6. Molecular Docking

**Figure 7:**
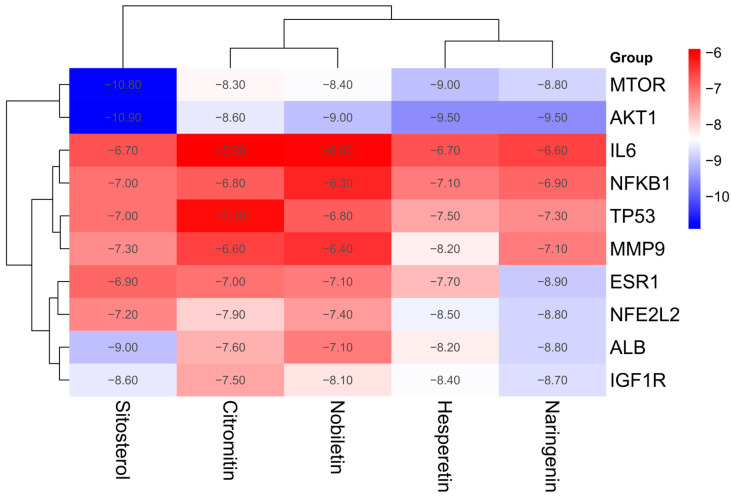
Heatmap illustrating the binding energies (kcal/mol) between the active ingredients of *Citri Reticulatae Pericarpium* and 10 hub targets (*n* = 50).

**Figure 8:**
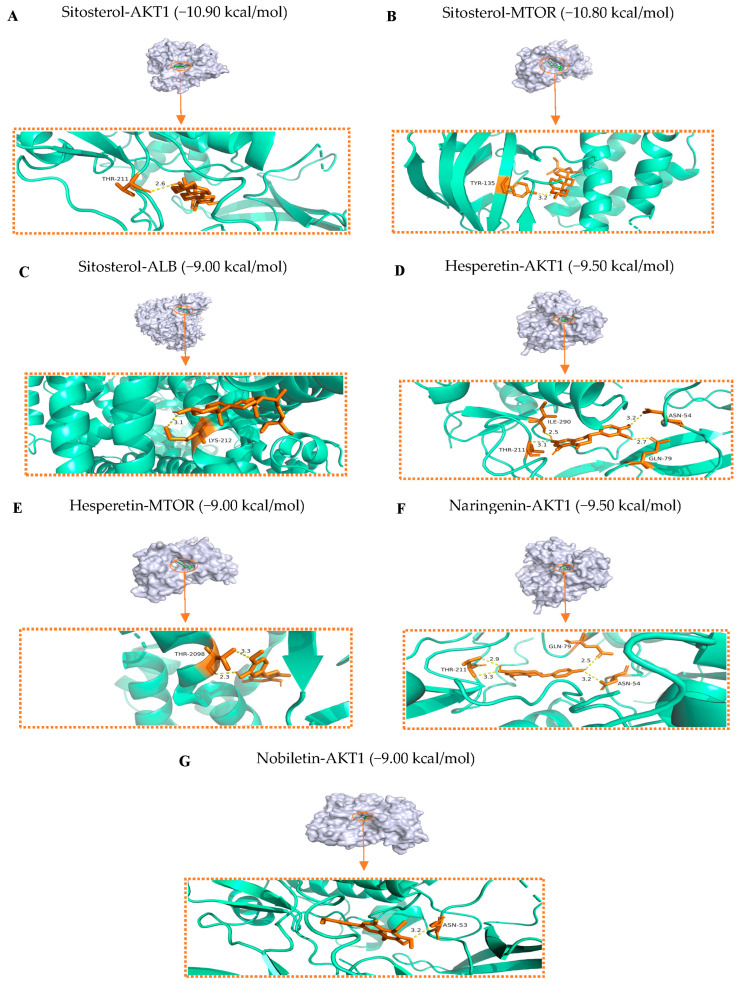
Molecular docking of Sitosterol, Hesperetin, Naringenin, and Nobiletin with AKT1, MTOR, and ALB proteins. (**A**) Sitosterol–AKT1; (**B**) Sitosterol–MTOR; (**C**) Sitosterol–ALB; (**D**) Hesperetin–AKT1, (**E**) Hesperetin–MTOR; (**F**) Naringenin–AKT1; (**G**) Nobiletin–AKT1.

A comprehensive understanding of the signaling pathways and interactions among the active ingredients and hub genes was achieved by docking the active ingredients (Sitosterol, Hesperetin, Naringenin, Nobiletin, and Citromitin) with AKT1 (PDB ID: 3o96), IL6 (PDB ID: 1alu), TP53 (PDB ID: 1kzy), MMP9 (PDB ID:1itv), ESR1 (1a52), NFKB1 (PDB ID: 8tqd), MTOR (PDB ID: 5gpg), IGF1R (PDB ID: 3d94), ALB (PDB ID: 1ao6), and NFE2L2 (PDB ID: 7x5f). The results showed that the active ingredients bound well to the hub genes, as indicated by their low binding energies, ranging from −10.90 to −5.90 kcal/mol (Figure 7). Lower binding energies usually produce more robust interaction probabilities and stable binding conformations. Binding energies below 0 kJ/mol indicate spontaneous binding, as suggested by previous studies. On the other hand, energies of −5.0 kJ/mol or lower indicate favorable binding activity [27]. Among the hub genes, AKT1 exhibited the highest binding affinity with the active ingredients, followed by MTOR, ALB, IGF1R, ESR1, NFE2L2, MMP9, TP53, NFKB1, and IL6. Sitosterol showed the highest docking score with the hub genes among the active ingredients. Hesperetin, Naringenin, Nobiletin, and Citromitin were the next ingredients with the highest docking scores. In contrast to the other groups, the binding efficiency of the AKT 1, MTOR, and ALB proteins, along with the three active ingredients, Sitosterol, Hesperetin, and Naringenin, revealed more excellent binding stability (Figure 8). The potential of these ingredients is evident by their lowest binding energies and successful attachment to the active regions of the hub genes. Overall, these findings suggest that these active ingredients can significantly alleviate sarcopenia through these potential targets.

### 2.7. Analysis of Molecular Dynamics Simulations

**Figure 9:**
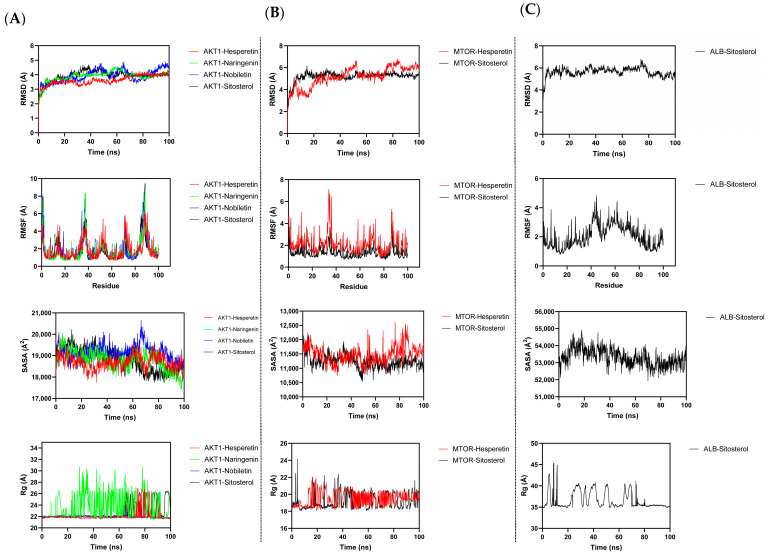
Molecular dynamics simulation of AKT1, MTOR, ALB, and active ingredients. (**A**) AKT1–Hesperetin/Naringenin/Nobiletin/Sitosterol; (**B**) MTOR–Hesperetin/Sitosterol; (**C**) ALB–Sitosterol.

Based on the docking results, the four best-performing active ingredients were selected for molecular dynamics simulations with AKT1 (PDB ID: 3o96), MTOR (PDB ID: 5gpg), and ALB (PDB ID: 1ao6) proteins. The protein–ligand complexes were assessed for stability using the root mean square deviation (RMSD). It is usually considered that the amplitude of variation declines and the stability of a protein increases as the RMSD value decreases. Among the complexes, the RMSD values of AKT1–Sitosterol and AKT1–Hesperetin fluctuated between 3 and 4 Å. However, the AKT1–Nobiletin, AKT1–Naringenin, MTOR–Hesperetin, MTOR–Sitosterol, and ALB–Sitosterol surpassed 4.5 Å in our analyses (Figure 9A–C). The AKT1–Sitosterol and AKT1–Hesperetin complexes initially fluctuated till 20 ns and showed a slight stabilization between 20 and 40 ns and ultimately stabilized at the final stages of simulation after 60 ns (Figure 9A). At the same time, the MTOR–Sitosterol complex fluctuated at the initial stages of simulation till 20 ns and showed stabilization after 20 ns till the final stages of simulation (Figure 9B). Root mean square fluctuation (RMSF) serves as an effective tool for evaluating the flexibility of proteins during MD simulation. The RMSF for the majority of the AKT1–Hesperetin, AKT1–Sitosterol, and MTOR–Sitosterol areas is below 2 Å, indicating the apparent flexibility of these proteins while bound to these ingredients (Figure 9A,B). For a more comprehensive understanding of the conformational stability of protein–ligand complexes, it is necessary to investigate solvent-accessible surface area (SASA), an important parameter in molecular dynamics simulation analysis. The SASA results indicated that AKT1–Hesperetin, AKT1–Sitosterol, and MTOR–Sitosterol complexes had a smooth fluctuation, suggesting that both the visible and covered regions of their surfaces experience minimal alteration and demonstrate enhanced binding affinity among the other complexes (Figure 9A–C). The radius of gyration (Rg) is used to evaluate the folding or unfolding of protein–ligand complexes during molecular dynamics simulation. It offers insights regarding the compactness of the complexes. The Rg analysis was used to evaluate the compactness of AKT1, MTOR, and ALB following the Hesperetin, Naringenin, Nobiletin, and Sitosterol binding. The results indicate that the complexes AKT1–Sitosterol, AKT1–Hesperetin, and MTOR–Sitosterol maintained compactness throughout the simulation process, with no significant changes compared to the other complexes (Figure 9A–C). The apo simulations of all the complexes can be found in the Supplementary Material, Figure S1.

### 2.8. Binding Free Energy MM-GBSA Calculations

The results of the binding free energy calculation of MM-GBSA showed that the AKT1–Sitosterol complex had the highest binding free energy of −102.22 kcal/mol, followed by ALB–Sitosterol, MTOR–Sitosterol, AKT1–Hesperetin, AKT1–Naringenin, AKT1–Nobiletin, and MTOR–Hesperetin with binding free energies of −91.12, −84.79, −62.47, −58.30, −58.30, and −27.14 kcal/mol, respectively (Table 3). The capacity of these molecules to bind with the target protein is signified by negative values, with more robust binding reflected by lower values. Our calculations revealed that MTOR–Hesperetin demonstrates a weak binding affinity, whereas the other small molecules had stronger binding affinity towards their respective proteins among all the complexes.

## 3. Discussion

Sarcopenia is the term used to describe the progressive decline in muscular mass and strength among aged adults, originating from the Greek terms “sarx,” denoting flesh, and “penia,” indicating loss. It is marked by a decline in skeletal muscle and reduced physical activity [28,29]. While sarcopenia usually develops with age, it can be accelerated by several mechanisms, such as lack of physical activity, inadequate nutrition, hormonal imbalances, age-related inflammatory processes, diminished blood flow, cellular senescence, and other features of aging biology [30]. The pathological process of sarcopenia is intricate, yet network pharmacology provides a distinct benefit in forecasting and elucidating the mechanism of action of traditional Chinese medicine (TCM). Despite the extensive use of network pharmacology in TCM, research on sarcopenia is limited and in its early stages [31]. Although there has been progress in research and therapeutic approaches, sarcopenia has not been widely acknowledged in therapeutic settings, and there is so far no pharmaceutical therapy available for this disease [32]. The prevention of sarcopenia will be facilitated by a more comprehensive understanding of its pathological, molecular, and identification mechanisms. Hence, the identification of novel pharmacological targets may provide prospects for the advancement of innovative therapeutic strategies for this condition. The complex nature of diseases poses challenges for the conventional “one disease-one target-one drug” model in Western medicine, limiting the identification of effective treatments for intricate or multiple conditions [33]. Considering the ability of TCM to target a wide range of diseases, it has gained significant popularity in contemporary research in recent years for treating conditions with intricate pathogenesis [34]. Given the intricate nature of sarcopenia, the present work utilized an in silico approach to explore the therapeutic potential of active ingredients of *Citri Reticulatae Pericarpium* in addressing sarcopenia through verification methods such as network pharmacology, molecular docking, and molecular dynamics simulation.

Hesperetin, Sitosterol, Citromitin, Naringenin, and Nobiletin were identified as the main active ingredients in CRP in this study. The identified active ingredients were also verified by a previous study through network pharmacology [35]. As mentioned earlier, sarcopenia is a degradation of skeletal muscle mass, reduced strength, and functionality resulting from old age. Dysfunctional mitochondria are now well acknowledged as a factor in age-associated muscle atrophy and functional decline. Hesperetin has been reported to reverse the age-related reduction in muscle fiber size and enhance running performance in a mouse model. Additionally, this compound enhanced mitochondrial reserve capacity by 25% and intracellular ATP by 33% [36]. Naringenin has been found to enhance skeletal muscle performance and inhibit muscle atrophy by improving the number of oxidative myofibers and promoting aerobic metabolism in a mouse model of muscular dystrophy [37]. Wang et al. demonstrated that the function and mass of skeletal muscle is enhanced by Nobiletin while improving myofiber size and the main protein composition of skeletal muscle in D-gal-induced aging mice [38]. The anticatabolic properties of β-sitosterol in skeletal muscles have been reported by Hah et al. Their results indicated that β-sitosterol suppressed muscle atrophy in animal models and cells treated with dexamethasone, a model of muscle atrophy caused by catabolism and a key contributing factor to aging sarcopenia [39]. The flavonoid Citromitin has been demonstrated to possess hepatoprotective properties [40], thereby protecting the liver against oxidative stress due to its antioxidant properties. Tsukamoto-Sen et al. indicated that regular antioxidant consumption could mitigate muscle function abnormalities associated with aging [41]. Citromitin may ameliorate muscle function due to its antioxidant properties. The potential of these active ingredients in treating sarcopenia was further validated by molecular docking and molecular dynamics simulation analyses. By targeting and modulating different pathways, these active ingredients may have a substantial impact in mitigating sarcopenia.

Following the network construction of the protein–protein interaction, AKT1, IL6, TP53, MMP9, ESR1, NFKB1, MTOR, IGF1R, ALB, and NFE2L2 were recognized as the top 10 hub genes by analyzing the 45 key intersecting targets in the protein–protein interaction network of *Citri Reticulatae Pericarpium* and sarcopenia. These hub genes may play a significant role by which active ingredients of *Citri Reticulatae Pericarpium* act in addressing sarcopenia. AKT1, MTOR, and ALB were the main and important targets found in our analysis. Among the hub genes, AKT1 was a crucial target, having the greatest degree node in the network. The protein AKT1 plays a significant role in numerous vital cellular processes, including normal and pathological events [42]. Sirago et al. highlighted the critical involvement of AKT1 in sarcopenia. They have shown that inhibition of AKT1 activity in skeletal muscle in a mouse model linked to insulin resistance and aging resulted in reduced skeletal muscle mass, disruption of motor function, and systemic insulin sensitivity. Therefore, its inhibition can accelerate osteosarcopenia and decrease lifespan [42]. Conversely, the activity of AKT1 may promote skeletal muscle mass and, hence, cope with age-related sarcopenia. MTOR was identified as the second most significant target in our analysis, followed by ALB. MTOR plays a crucial role in controlling muscle anabolic and catabolic pathways, making it a potential therapeutic target for combating sarcopenia [43]. The synthesis of muscle proteins and myogenesis via activation of the AKT/mTOR signaling pathway has been reported by Oh et al. in a sarcopenic mouse model [44]. The protein albumin (ALB), commonly synthesized by liver hepatocytes, is the predominant protein found abundantly in the plasma of healthy human adults [45]. An elevated risk of functionality impairment and decreased muscle mass is linked to low levels of ALB protein [46]. These genes may serve as the possible targets for the active ingredients of *Citri Reticulatae Pericarpium* in addressing sarcopenia.

The active ingredients of *Citri Reticulatae Pericarpium* and their potential therapeutic effect in treating sarcopenia were further supported by the enrichment analysis. The gene ontology results indicated that responses to peptides, peptide hormones, and extracellular stimuli were the main biological processes. In contrast, the main cellular components and molecular functions such as ficolin-1-rich granule lumen, secretory granule lumen, cytoplasmic vesicle lumen, estrogen response element binding, nuclear factor receptor activity, and ligand-activated transcription factor activity were respectively enriched. The Kyoto Encyclopedia of Genes and Genomes analysis revealed that PIK3-Akt, HIF-1, and longevity-regulating pathways were involved in the therapeutic role of the active ingredients in treating sarcopenia. Several cellular activities in vivo are mediated by the PI3K/Akt signaling pathway, which is one of the key signal transduction pathways [46]. The PI3K/Akt signaling pathway can promote the proliferation and differentiation of myogenic cells. It also boosts skeletal muscle development [47]. The master regulator of oxygen homeostasis, the HIF-1 signaling pathway, regulates oxidative stress, injury, angiogenesis, vascular remodeling, inflammatory reactions, and metabolic remodeling [48]. Mitochondrial biogenesis and oxidative metabolism are impaired by HIF-1 during sarcopenia, resulting in a decline in muscle mass function [49]. Inhibition of the HIF-1 signaling pathway may thereby ameliorate sarcopenia [4]. The longevity-regulating pathway plays a significant role in the aging process [50]. Wu et al. have found that lactoferrin and creatine inhibit the progression of sarcopenia by modulating the quantity and cross-sectional area of muscle fibers, and muscle protein synthesis, together with significant enrichment in the longevity regulating pathway [51].

The findings were further validated by molecular docking and molecular dynamics simulations analyses. The active ingredients Hesperetin, Sitosterol, Naringenin, Citromitin, and Nobiletin revealed a notable binding affinity varied between −10.90 and −5.80 kcal/mol with the hub genes. Spontaneous binding is indicated by binding energies less than 0 kJ/mol while binding energies of −5.0 kJ/mol or less indicate favorable binding activity [27]. The lowest binding energies of AKT1, MTOR, and ALB were the best among the hub genes. The docking analysis of the ingredients Hesperetin, Naringenin, Nobiletin, and Sitosterol with the AKT1, MTOR, and ALB proteins was performed based on the binding scores. The docking predictions demonstrate that these active ingredients effectively bind to the active regions of the hub genes, indicating that these ingredients can efficiently exploit these prospective targets to alleviate sarcopenia. The interactions and binding between these active ingredients and prospective targets were further validated by molecular dynamics simulation analysis. The complexes Hesperetin–AKT1 (PDB ID: 3o96), Hesperetin–MTOR (PDB ID: 5gpg), Naringenin–AKT1 (PDB ID: 3o96), Nobiletin–AKT1 (PDB ID: 3o96), Sitosterol–AKT1 (PDB ID: 3o96), Sitosterol–MTOR (PDB ID: 5gpg), and Sitosterol–ALB (PDB ID: 1ao6) were subjected to simulation. The average binding energy of the selected complexes for simulation was −9.67 kcal/mol. Through the formation of high interactions between a ligand and a receptor, the stabilization of a protein–ligand complex is significantly influenced by hydrogen bonds. They are crucial in determining the specificity, metabolism, and absorption of drugs used in drug design [52]. Hesperetin was observed to interact with AKT1 protein through THR-211, ILE-290, ASN-54, and GLN-79 amino acid residues and formed four hydrogen bonds. It interacts with MTOR protein via THR-2098 amino acid residue, forming two hydrogen bonds. Naringenin formed four hydrogen bonds with AKT1 through THR-211, GLN-79, and ASN-54 amino acid residues. Nobiletin interacts with AKT1 through ASN-53, forming one hydrogen bond. The interaction of Sitosterol with AKT1, MTOR, and ALB proteins was observed via THR-211, TYR-135, and LYS-212 amino acid residues while it formed a single hydrogen bond with these proteins. Among all the complexes, Hesperetin–AKT1 (3o90) and Naringenin–AKT1 (3o90) formed four hydrogen bonds, followed by Hesperetin–MTOR (5gpg) with two bonds, and Nobiletin–AKT1 (3o90), Sitosterol–AKT1 (3o90), Sitosterol–MTOR (5gpg), and Sitosterol–ALB (1ao6) with a single hydrogen bond, respectively. These compounds exhibit strong binding and interactions with the AKT1, MTOR, and ALB proteins, as shown by hydrogen bonding analysis, wherein they effectively interact with various amino acids. The structural integrity of these proteins is particularly evident when they are bound to these active ingredients. Furthermore, at a temperature of 300 K, these complexes exhibited structural stability during molecular dynamics simulation. Particularly, the stability of AKT1-Sitosterol, AKT1–Hesperetin and MTOR-Sitosterol was apparent as compared to the other complexes. The therapeutic potential of these compounds in combating sarcopenia was demonstrated by their strong affinity and structural stability toward the relevant targets. In order to further validate the findings of this in silico study, in vitro and in vivo trials are warranted.

Our study has some limitations. First, in silico studies are usually computer-based predictions; therefore, experimental verification is necessary to confirm our findings. Second, the in silico approach has limitations in thoroughly evaluating the specific role and understanding the underlying mechanism by which the active ingredients of *Citri Reticulatae Pericarpium* are used in addressing sarcopenia. However, our study provides the basis for future trials and potential new ingredients for sarcopenia treatment.

## 4. Materials and Methods

### 4.1. Software and Databases

The present in silico investigation utilized various databases and software to collect data on *Citri Reticulatae Pericarpium* and sarcopenia to evaluate the efficacy of the active ingredients of *Citri Reticulatae Pericarpium* in addressing sarcopenia.

### 4.2. Database Construction for Active Ingredients

A database containing five active ingredients derived from *Citri Reticulatae Pericarpium* was created. The three-dimensional (3D) structures of these ingredients, along with the canonical SMILES, were retrieved using the PubChem registry by querying the terms “Hesperetin”, “Naringenin”, “Nobiletin”, “Sitosterol”, and “Citromitin”. We selected the most suitable matches for these ingredients, and the obtained 3D structures were stored in SDF format for subsequent investigation. Drug repurposing and virtual screening are among the most prevalent applications of PubChem^®^ (https://pubchem.ncbi.nlm.nih.gov) (accessed on 18 August 2024), a chemical database maintained by the National Library of Medicine. The active ingredients Hesperetin, Nobiletin, Naringenin, Citromitin, and Sitosterol from *Citri Reticulatae Pericarpium* were the primary ingredients investigated in this study for addressing sarcopenia.

### 4.3. Identification of Active Ingredients of Citri Reticulatae Pericarpium and Prospective Targets

The Traditional Chinese Medicine Systems Pharmacology (TCMSP) is an integrated pharmacological interface for Chinese medicinal herbs, providing interactive data on the relationships among medications, targets, and disorders [53]. The prospective targets and active ingredients of *Citri Reticulatae Pericarpium* were identified and acquired through an extensive search in the TCMSP platform (https://tcmsp-e.com/) (accessed on 17 August 2024). The entries in the platform can be sorted by pharmacokinetic criteria, including drug absorption, distribution, metabolism, and excretion (ADME) [54]. The TCMSP platform was searched for the active ingredients utilizing the search terms “Citrus reticulata” or “Chenpi” as the herbal identity. To evaluate the active ingredients, the oral bioavailability (OB) and drug-likeness (DL) parameters were established at ≥30% and ≥0.18 [12]. The prospective targets of these ingredients were subsequently retrieved from the platform. By setting the screening parameters of a druggable score of ≥0.1 and a confidence score of ≥0.95, a comprehensive search was performed in the BATMAN-TCM database (http://bionet.ncpsb.org.cn/batman-tcm/) (accessed on 18 August 2024), a bioinformatics annotation tool designed to elucidate the molecular processes of TCM. The BATMAN-TCM database is extensively employed in TCM to assess the effect targets of active ingredients [55]. Additionally, the PharmMapper (https://www.lilab-ecust.cn/pharmmapper/submitfile.html) (accessed on 18 August 2024) database was utilized to identify additional targets for these active ingredients [56]. All targets from BindingDB, DrugBank, PDTD, and TargetBank are incorporated into the extensive internal registry of pharmacophore databases that support the PharmMapper database [57]. More predicted targets of the identified active ingredients were achieved by entering their canonical SMILES into the search engine of the prediction (https://prediction.charite.de/) (accessed on 18 August 2024) database [58]. Subsequently, the targets were transformed into gene symbols using the Uniprot (https://www.uniprot.org) (accessed on 18 August 2024) database [59]. All the pooled targets from various sources were compiled, and any repeated entries were eliminated.

### 4.4. Target Acquisition of Sarcopenia

Six databases were utilized to retrieve sarcopenia-related targets. Initially, prospective targets of sarcopenia were identified by conducting a search term “sarcopenia” in the GeneCards (https://www.genecards.org/) (accessed on 19 August 2024) database [60]. To obtain a pool of more potential targets, the Online Mendelian Inheritance in Man (OMIM) (https://www.omim.org/) (accessed on 19 August 2024) [61], PharmGKB (https://www.pharmgkb.org/) (accessed on 19 August 2024) [62], DISGENET (https://www.disgenet.org/) (accessed on 19 August 2024) [63], National Center for Biotechnology Information (NCBI) (https://www.ncbi.nlm.nih.gov/) (accessed on 20 August 2024) [64], and Comparative Toxicogenomics Database (CTD) (http://ctdbase.org/) (accessed on 20 August 2024) databases [65] were queried, respectively. The obtained targets were then combined, and the duplicate genes were eliminated.

### 4.5. Key Intersecting Targets of Citri Reticulatae Pericarpium and Sarcopenia

To identify the key intersecting targets between *Citri Reticulatae Pericarpium* and sarcopenia, the prospective targets of the active ingredients of *Citri Reticulatae Pericarpium* and sarcopenia were submitted to the online platform (http://www.interactivenn.net/) (accessed on 21 August 2024) [66]. Subsequently, a Venn diagram between *Citri Reticulatae Pericarpium* and sarcopenia was generated.

### 4.6. Protein–Protein Interaction Network Generation

The intersected core targets of *Citri Reticulatae Pericarpium* and sarcopenia were analyzed using the online STRING (https://string-db.org/) (accessed on 21 August 2024) database version 12.0, resulting in the generation of the protein–protein interaction network. The organism “Homo sapiens” and the search criterion “Multiple proteins” were employed to search the STRING platform. The system configuration was determined to be the minimal interaction score with medium confidence of “0.400”, and the configuration option to “hide disconnecting nodes in the network” was chosen [67]. For all other parameters, the default configurations of the system were unchanged. The TSV file containing the generated protein–protein interaction network was then exported to Cytoscape software 3.9.1. A subsequent analysis and visualization of the network was conducted with Cytoscape 3.9.1. The top 10 hub genes were identified by utilizing the cytoHubba extension tool of Cytoscape software 3.9.1. The maximal clique centrality (MCC) of the top 10 nodes was the basis for selecting the parameters used in this calculation.

### 4.7. Enrichment Analysis of Gene Ontology and Kyoto Encyclopedia of Genes and Genomes Pathway

The Metascape database is an efficient framework for performing thorough analyses and interpretations of omics in the context of big data. It offers various functionalities, including gene annotation, interactive analysis, and functional enrichment. Incorporating the Kyoto Encyclopedia of Genes and Genomes and Gene Ontology enrichment analysis while discovering drug–disease signaling networks facilitates an extensive comprehension of the functionality of significant genes [68]. By integrating the key intersecting genes from (http://www.interactivenn.net/) (accessed on 21 August 2024) into the Metascape (https://metascape.org/) (accessed on 22 August 2024) platform, the Gene Ontology and Kyoto Encyclopedia of Genes and Genomes analyses were conducted. The results of the enrichment analysis for biological processes, cellular components, molecular functions, and Kyoto Encyclopedia of Genes and Genomes pathways were acquired. The database setup was designated as the “gene list” type, specifying the “Homo sapiens” species. An enrichment analysis was performed with a *p*-value of <0.01, an enrichment value > 1.5, and an optimal overlap of 3. The online bioinformatics (https://www.bioinformatics.com.cn/en) (accessed on 22 August 2024) mapping platform was used to create the enrichment bubble charts and bar graphs by importing the top 20 Kyoto Encyclopedia of Genes and Genomes pathways and top 10 gene ontology terms into the platform according to their *p*-values [69].

### 4.8. Herb-Compound-Target-Pathway Network Construction

The top 20 Kyoto Encyclopedia of Genes and Genomes pathways, their associated targets, and the active ingredients and *Citri Reticulatae Pericarpium* were imported from the enrichment analysis to construct the herb-compound-target-pathway interaction network in Cytoscape 3.9.1. The software subsequently visualized the herb-compound-target-pathway network of the active ingredients of *Citri Reticulatae Pericarpium* and sarcopenia, and a graphical diagram was generated.

### 4.9. Molecular Docking

Molecular docking is a method that tends to significantly forecast the efficacy and interaction procedures of active ingredients with prospective targets by creating interactions between small ligands and related targets [70]. The 3D conformer of the active ingredients anticipated to interact with the hub genes was retrieved from the (https://pubchem.ncbi.nlm.nih.gov/) (accessed on 18 August 2024) database and saved as SDF formats. Following the importation of the SDF file containing the 3D structures of the active ingredients into Chem3D software 12.0.2, they underwent an energy minimization process and were exported as mol2 format for further analysis. Furthermore, the 3D and crystal X-ray conformers utilized in prior investigations were obtained from the UniProt and the Worldwide Protein Data Bank (RCSBPDB) (https://www.rcsb.org/) (accessed on 22 August 2024) database. Entities corresponding to “Homo sapiens” species were chosen specifically based on resolutions ranging from 2.0 to 3.0 Å [71]. Consequently, the 3D crystal structures of AKT1 (PDB ID: 3o96), IL6 (PDB ID: 1alu), TP53 (PDB ID: 1kzy), MMP9 (PDB ID:1itv), ESR1 (1a52), NFKB1 (PDB ID: 8tqd), MTOR (PDB ID: 5gpg), IGF1R (PDB ID: 3d94), ALB (PDB ID: 1ao6), and NFE2L2 (PDB ID: 7x5f) were retrieved from RCSBPDB database. PyMol 2.5.8 was utilized to eliminate water, and ligand molecules were removed from the protein structures. The protein structures were subsequently imported into AutoDock Tools 1.5.6, where non-polar hydrogen atoms were assigned, and the resulting structures were transformed into a PDBQT file. In addition, the small molecule ligands (Hesperetin, Naringenin, Nobiletin, Sitosterol, and Citromitin) were converted to the PDBQT format to facilitate docking operations. Ultimately, docking operation was conducted using Autodock Vina software 1.5.6. PyMol 2.5.8 was used to visualize the interaction between the active ingredients and hub genes, and subsequent docking and pocket models were generated. PyMOL 2.5.8 is an open-source software package intended for the visualization of molecular models. It can identify hydrogen bonds and assess the binding affinity of receptor proteins and ligands in molecular docking research [54].

### 4.10. Molecular Dynamics Simulation

The drug design approach frequently involves molecular dynamics simulation, which is essential for understanding proteins’ dynamic procedures and structural changes [72]. Molecular dynamics simulation was performed to examine the binding affinities of the active ingredients and target proteins determined by molecular docking. Molecular docking studies offer predictions regarding ligand binding under static environments. It provides a static representation of a molecule’s binding conformation within a protein’s active site, whereas molecular dynamics simulation calculates atomic movements over time by integrating Newton’s classical equations of motion. The molecular dynamics simulations were performed for 100 ns using Desmond software 2019-4, a Package by Schrödinger LLC [73]. The preliminary structures of protein–ligand complexes were derived from docking analyses to facilitate the simulation operation. The ligand binding status in the physiological environment was predicted by integrating Newton’s classical equation of motion by molecular dynamics simulation. The protein–ligand complexes were optimized and minimized during the preprocessing procedure using Protein Preparation Wizard or Maestro [74]. All systems were configured using the System Builder tool. The TIP3P (Transferable Intermolecular Interaction Potential 3 Points) was determined to be the solvent model with an orthorhombic box [75]. The OPLS_2005 force field was used in the simulation [76]. The models were made neutral by adding counter ions where needed, and 0.15 M salt (NaCl) was added to mimic the physiological conditions. The NPT ensemble was chosen for the entire simulation, with a temperature of 300 K and a pressure of 1 atm. The Nose–Hoover Chain (NHC) Thermostat and Martyna–Tuckerman–Klein (MTK) Barostat were used for an NPT ensemble [77,78]. The Particle Mesh Ewald (PME) was used to calculate the Coulombic interactions [79,80]. The SHAKE algorithm was employed to constrain hydrogen atom bonding [81]. Before the simulation, the models were subjected to relaxation. The stability of the simulations was assessed by calculating the root mean square deviation (RMSD) of the protein and ligand over time, and the trajectories were recorded every 10 ps for analysis.

Furthermore, the MM-GBSA binding free energies were calculated using the thermal_mmgbsa.py Python script released by Schrodinger. This script also calculates coulomb energy, covalent binding energy, van der Waals energy, lipophilic energy, generalized Born electrostatic solvation energy, and hydrogen-bonding energy. The binding energies were calculated using the following equations:

Gbind = ∆E_MM_ + ∆G_solv_ + ∆G_SA_ (1)

where ΔEMM represents the difference in minimized energies among the complex, ligand, and protein, as follows:

∆E_MM_ = ΔG_Coulomb_ + ΔG_Covalent_ + ΔG_H-bond_ + ΔG_Lipo_ + ΔG_vdW_ (2)

The term ΔG_solv_ represents the difference between the GBSA solvation energy of the complex and the sum of the solvation energies of the ligand and protein, whereas ΔGSA represents the difference between the surface area energy of the complex and the sum of the protein and ligand.

## 5. Conclusions

The current in silico study identified the active ingredients of *Citri Reticulatae Pericarpium*, prospective targets, and pathways and explored the therapeutic potential and significant role of these ingredients in treating sarcopenia. Five active ingredients with 45 core intersecting targets between *Citri Reticulatae Pericarpium* and sarcopenia were found. AKT1, IL6, TP53, MMP9, ESR1, NFKB1, MTOR, IGF1R, ALB, and NFE2L2 were the top 10 hub targets found in the protein–protein interaction network. Our findings revealed that AKT1 and MTOR are the crucial targets by which the active ingredients of *Citri Reticulatae Pericarpium*, particularly Sitosterol and Hesperetin, may act to alleviate sarcopenia through the PI3K-AKT signaling pathway. Furthermore, the significant role of these active ingredients against sarcopenia may involve the HIF-1 signaling pathway and longevity regulating pathway. The present study provided significant insights into the therapeutic potential of the active ingredients of *Citri Reticulatae Pericarpium* for treating sarcopenia. However, the therapeutic effect of these ingredients against sarcopenia requires confirmation by in vitro and in vivo trials.

## Figures and Tables

**Table 1:** Software and databases used in this study.

Software/Database	Version	Website
Cytoscape	3.9.1	https://cytoscape.org/ (accessed on 21 August 2024)
Chem3D	12.0.2	https://revvitysignals.com/ (accessed on 18 August 2024)
AutoDockTools	1.5.6	http://mgltools.scripps.edu/ (accessed on 22 August 2024)
PyMol	2.5.8	https://pymol.org/ (accessed on 23 August 2024)
PubChem	1.8.0 beta	https://pubchem.ncbi.nlm.nih.gov/ (accessed on 18 August 2024)
TCMSP	2.3	https://old.tcmsp-e.com/tcmsp.php (accessed on 17 August 2024)
BATMAN-TCM	2.0	http://bionet.ncpsb.org.cn/batman-tcm/#/home (accessed on 18 August 2024)
PharmMapper	2017	https://www.lilab-ecust.cn/pharmmapper/ (accessed on 18 August 2024)
Prediction	2022	https://prediction.charite.de/ (accessed on 18 August 2024)
UniProt	2024.4	https://www.uniprot.org/ (accessed on 18 August 2024)
GeneCards	5.21.0	https://www.genecards.org/ (accessed on 19 August 2024)
OMIM	2024.8.26	https://omim.org/ (accessed on 19 August 2024)
PharmGKB	4.0	https://www.pharmgkb.org/ (accessed on 19 August 2024)
DISGENET	24.2	https://disgenet.com/ (accessed on 19 August 2024)
NCBI	262.0	https://www.ncbi.nlm.nih.gov/ (accessed on 20 August 2024)
CTD	2024.7.31	https://ctdbase.org/ (accessed on 20 August 2024)
InteractiVenn	2015	https://www.interactivenn.net/ (accessed on 21 August 2024)
STRING	12.0	https://cn.string-db.org/ (accessed on 21 August 2024)
Metascape	3.5.20240101	https://metascape.org/ (accessed on 22 August 2024 )
Wei Sheng Xin	2024	https://www.bioinformatics.com.cn/ (accessed on 22 August 2024)
RCSBPDB	2024.8.27	https://www.rcsb.org/ (accessed on 22 August 2024)
Desmond	2019-4	https://www.schrodinger.com/ (accessed on 26 August 2024)

TCMSP: Traditional Chinese Medicine Systems Pharmacology; BATMAN-TCM: Bioinformatics Annotation daTabase for Molecular mechANism of Traditional Chinese Medicine; OMIM: Online Mendelian Inheritance in Man; NCBI: National Center for Biotechnology Information; CTD: Comparative Toxicogenomics Database; RCSBPDB: Protein Data Bank.

**Table 2:** Properties of the active ingredients in *Citri Reticulatae Pericarpium*.

Molecule ID	Molecule Name	OB (%)	DL	Molecular Formula	CAS Number	Molecular Weight (g/mol)
MOL005100	Hesperetin	47.74	0.27	C_16_H_14_O_6_	520-33-2	302.28
MOL004328	Naringenin	59.29	0.21	C_15_H_12_O_5_	480-41-1	272.25
MOL005828	Nobiletin	61.67	0.52	C_21_H_22_O_8_	478-01-3	402.4
MOL000359	Sitosterol	36.91	0.75	C_29_H_50_O	83-46-5	414.7
MOL005815	Citromitin	86.90	0.51	C_21_H_24_O_8_	3570-71-6	404.4

**Table 3:** Calculated MM-GBSA binding free energies (kcal/mol).

Complex Name	ΔG_bind_	ΔG_Coulomb_	ΔG_Covalent_	ΔG_H-bond_	ΔG_Lipo_	ΔG_Solv_GB_	ΔG_SA_	ΔG_vdW_
AKT1–Hesperetin	−62.47	−4.97	8.92	−0.54	−50.93	21.61	−0.25	−36.31
AKT1–Naringenin	−58.30	−6.14	5.44	−0.25	−47.22	26.61	−0.23	−36.50
AKT1–Nobiletin	−58.30	−6.14	5.44	−0.25	−47.22	26.61	−0.23	−36.50
AKT1–Sitosterol	−102.22	−11.26	2.44	−0.49	−65.86	30.91	−0.32	−57.63
MTOR–Hesperetin	−27.14	−1.21	1.96	−0.52	−16.16	8.01	−0.08	−19.14
MTOR–Sitosterol	−84.79	−0.66	2.09	−0.02	−61.97	28.05	−0.31	−51.97
ALB–Sitosterol	−91.12	−4.89	2.81	−0.08	−54.51	10.86	−0.27	−45.04

ΔG_bind_: free energy of binding; ΔG_coulomb_: Coulomb energy of the complex; ΔG_Covalent_: covalent energy; ΔG_H-bond_: hydrogen-bonding; ΔG_Lipo_: lipophilic energy; ΔG_Solv_GB_: generalized Born electrostatic solvation energy; ΔG_SA_: nonpolar solvation energy; ΔG_VdW_: van der Waals energy.

## Data Availability

The original contributions presented in the study are included in the article/Supplementary Material, further inquiries can be directed to the corresponding authors.

## Supplementary Materials

The following supporting information can be downloaded at: https://www.mdpi.com/article/10.3390/ijms252111451/s1.
