# Supplementary material for: Insights into the Therapeutic Potential of Active Ingredients of Citri Reticulatae Pericarpium in Combatting Sarcopenia: An In Silico Approach
Source: Int J Mol Sci. 2024 Oct 25;25(21):11451. doi: 10.3390/ijms252111451 (PMC11546236; doi:10.3390/ijms252111451)
Supplement: Supplementary file 1 [file ijms-25-11451-s001.zip › ijms-3261052-supplementary.pdf]

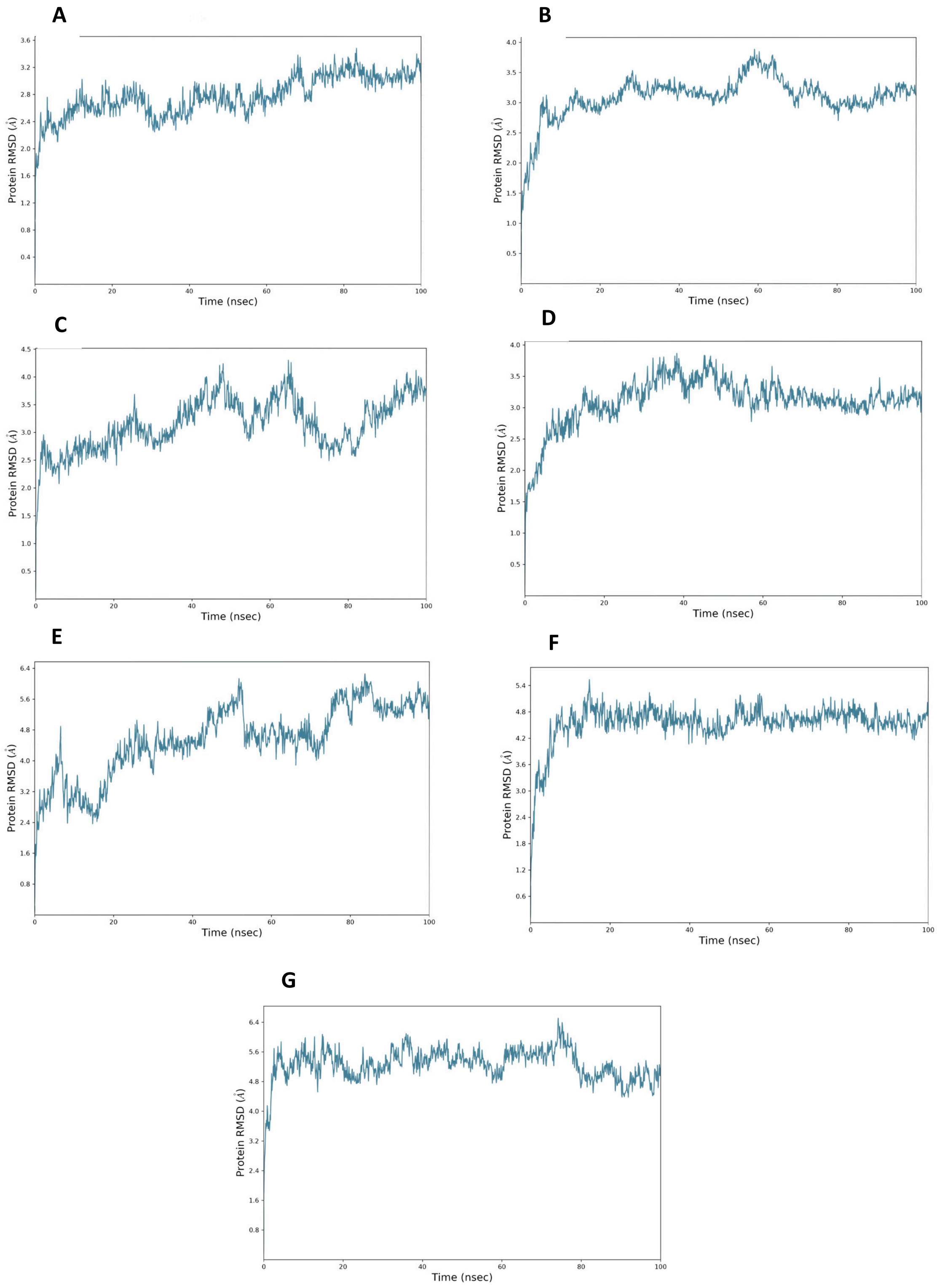

Figure S1. Apo simulations of all complexes. (**A**) AKT1/Hesperetin; (B) AKT1/Naringenin; (C) AKT1/Nobiletin; (D) AKT1/Sitosterol; (E) MTOR/Hesperetin; (F) MTOR/Sitosterol; (G) ALB/Sitosterol
